# Variations of plutonium isotopic ratios in Antarctic ecosystems

**DOI:** 10.1007/s10967-018-6274-6

**Published:** 2018-10-29

**Authors:** Katarzyna M. Szufa, Jerzy W. Mietelski, Robert Anczkiewicz, Dariusz Sala, Maria A. Olech

**Affiliations:** 1 Institute of Nuclear Physics, Polish Academy of Sciences, Radzikowskiego 152, 31-342 Kraków, Poland; 2 Institute of Geological Sciences, Research Centre in Kraków, Polish Academy of Sciences, Senacka 1, 31-002 Kraków, Poland; 3 Institute of Botany, Jagiellonian University, Gronostajowa 3, 30-387 Kraków, Poland; 4 Department of Antarctic Biology, Institute of Biochemistry and Biophysics, Polish Academy of Sciences, Pawinskiego 5a, 02-106 Warszawa, Poland

**Keywords:** Plutonium ratios, Antarctica, Marine versus terrestrial ecosystems, Inductively coupled plasma mass spectrometry (ICP-MS), Alpha spectrometry

## Abstract

The aim of the article was to verify the hypothesis concerning the diversification of plutonium sources in the natural environment of Antarctica. Plutonium activity and atom ratios were analyzed in two groups of biological samples: terrestrial and marine. Both isotopic ratios in the terrestrial set were consistent with global radioactive fallout ratios. The average activity ratio in the marine ecosystem was lower than global radioactive fallout. At the same time mass ratio values in this group turned out to be surprisingly varied. Analysis of the results showed statistically significant differences between the marine and terrestrial ecosystems.

**Electronic supplementary material:**

The online version of this article (10.1007/s10967-018-6274-6) contains supplementary material, which is available to authorized users.

## Introduction

Antarctica is an isolated continent with unique and little-varied ecosystems. Due to its location and separation by cold oceanic waters, it is a good study area to analyze radionuclides’ transport in the environment. Radioactive debris injected into the stratosphere during the atmospheric nuclear weapons tests and accidents spread around the globe reaching Antarctica as well. Some research has been conducted on radioactivity in Antarctica [1–7] which has pointed out that global fallout with significant input of ^238^Pu from the “SPAP-9A” accident is a source of Antarctic radioactive contamination. There were suggestions that there is a difference between marine and terrestrial samples in ^238^Pu/^239+240^Pu activity ratios [5]. It was not investigated further in the past because of sparse data. However, a hypothesis about the influence of sources of different plutonium ratios on marine and terrestrial ecosystems was put forward. The present article is aimed to study Antarctic marine and terrestrial ecosystems to check the hypothesis about different plutonium sources in the Antarctic environment. To achieve this goal, the biological samples were analyzed sequentially by alpha spectrometry and inductively-coupled mass spectrometry (ICP-MS) methods.

## Experimental

**Fig. 1:**
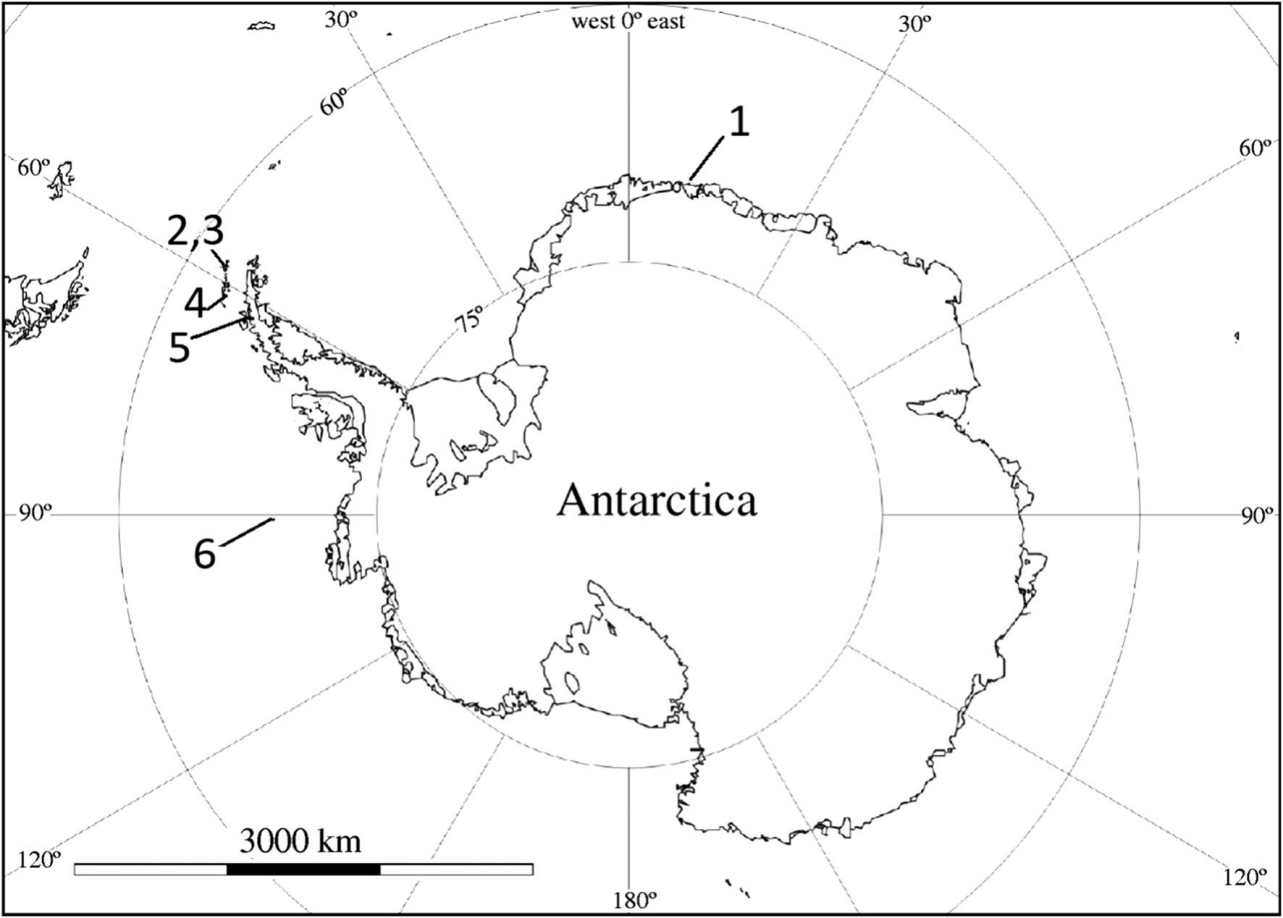
Sampling area. 1—Schirmacher Oasis, 2, 3—King George Island and Penguin Island, 4—Deception Island, 5—Antarctic Peninsula (Brown Station), 6—Peter I Island

Fifty nine samples from the marine environment and fifty two samples from the terrestrial environment were investigated. During a series of Polish Academy of Sciences expeditions carried out over three decades (1980–2015) research material was collected. It includes: bones, feathers, soft tissues and egg shells of *Pygoscelis adeliae*, feathers of *Pygoscelis papua*, bones, feathers, soft tissues of *Macronectes giganteus, Pagodroma nivea* and *Catharacta antarctica*, bones, skin and fur of *Leptonychotes weddellii*, skin and fur of *Mirounga leonina*, soft tissue and shells *of Nacella concina*, fish *Harpagifer antarcticus* and *Chaenocephalus aceratus*, algae *Himantothallus grandifolius* and *Iridaea cordata* from marine environment and lichens *Usnea antarctica*, *Usnea aurantiaco*-*atra*, moss *Sanionia uncinata,* grass *Deschampsia antarctica*, initial soils from terrestrial environment. Birds were classified as marine organisms by reason of their hunting and eating habits and the long time intervals they spend at sea (only breeding on the land). The majority of samples was obtained on King George Island (South Shetland Islands) while the rest was collected on Deception Island (South Shetland Islands), Penguin Island (South Shetland Islands), Peter I Island and continental places (Schirmacher Oasis, Antarctic Peninsula). Detailed information can be found in Tables S1-S5 in the supplementary data and map presenting sampling sites (Fig. 1). Animals were not obtained as live specimens; only remains of the animals’ bodies were collected *post mortem*.

Samples were ashed at 600 °C (previously, bird and mammal samples were ashed at 400 °C after which bones and soft tissues were separated) and following this operation, ^242^Pu tracer (NIST attested) was added by weight. Bones, eggs and mollusk shells were dissolved in 9 M HCl, and at pH ~ 3, precipitate of calcium oxalates containing plutonium isotopes were performed. The procedure is described in detail elsewhere [8–10]. The rest of the samples was mineralized using hot, concentrated acids (40% HF, 65% HNO_3_, 35% HCl) with subsequent addition of H_3_BO_3_ to complex fluoride. Plutonium isotopes were isolated using anion exchange chromatography using DOWEX 1 × 8 [11–14] after adjustment to the + 4 oxidation state of Pu using hydrazine and NaNO_2_ [11]. Alpha spectrometric sources were prepared by NdF_3_ micro coprecipitation method [15] using 100 nm pore diameter membrane filters by Eichrom. The average chemical recovery was 76% with standard deviation 17%; minimal was 20% (in 111 sample set, for 5 samples chemical recovery was below 50%) and maximal 100%. Measurements of ^238,239+240^Pu were performed using different alpha spectrometers (Silena Alpha Quattro, Ortec Alpha Duo or Canberra 7401); all were equipped with Canberra or EG&G Ortec ion-implanted silicon detectors of 450 mm^2^ area each with about 2 mm source-detector distance. After determining ^238^Pu and ^239+240^Pu activity concentrations in samples, 44 of them (i.e., those which have Pu above detection limit in alpha spectrometric measurement) were chosen and prepared for the mass spectrometry measurements. Plastic membrane filters loaded with NdF_3_ precipitate were dissolved using HNO_3_, HCl, H_3_BO_3_ and HClO_4_ as it is described in [16, 17]. The Pu oxidation state was again adjusted to + 4 according to the above-mentioned procedure, setting the final solution to 4 M HNO_3_. Then samples were purified from uranium and thorium traces by passing through TEVA resin [16, 17]. Plutonium fractions of all samples except bones and soft tissues were evaporated to salts. Bone and soft tissue samples went through purification on DOWEX 1 × 8, Pu previously being adjusted to + 4. This was done to remove any traces of phosphates. Those plutonium effluents were evaporated as well. The last stage of preparation to ICP-MS measurement was to dissolve salts obtained from evaporation in 2% HNO_3_—0.1% HF. Such solutions were introduced to mass spectrometry analysis.

## Results and discussion

For the purpose of describing the results, marine samples were divided into four groups:

1. soft tissues and feathers of birds,
2. bones of birds,
3. algae,
4. other organisms.

Terrestrial samples were divided into three groups:

1. mosses,
2. lichens,
3. others (grass or soil).

Statistical parameters which characterize results are presented in Table 1. All results are set in supplementary data Tables S6 and S7. Pu activity concentrations in marine samples are on basis of ash weight, in terrestrial samples of dry weight.

**Table 1:** Percentage of results above detection limits, arithmetic mean with standard deviation, minimal and maximal obtained in Bq/kg and, mean with standard deviation, minimal and maximal of ^238^Pu/^239+240^Pu activity ratio and ^240^Pu/^239^Pu atom ratio values

	^238^Pu			^239+240^Pu			^238^Pu/^239+240^Pu		^240^Pu/^239^Pu	
	C > LD. %	Mean ± SD	Min max	C > LD. %	Mean ± SD	Min max	Mean ± SD	Min max	Mean ± SD	Min max
Terrestrial ecosystem	79	0.13 ± 0.14	0.0058 ± 0.0009 0.55 ± 0.10	94	0.62 ± 0.81	0.0010 ± 0.0002 4.00 ± 0.34	0.17 ± 0.03	0.08 ± 0.01 0.27 ± 0.05	0.17 ± 0.01	0.147 ± 0.001 0.192 ± 0.001
Mosses	78	0.10 ± 0.13	0.0058 ± 0.0009 0.55 ± 0.10	96	0.47 ± 0.72	0.0010 ± 0.0002 3.33 ± 0.31	0.17 ± 0.04	0.08 ± 0. 0.27 ± 0.05		
Lichens	90	0.16 ± 0.14	0.021 ± 0.003 0.55 ± 0.10	95	0.86 ± 0.91	0.018 ± 0.003 4.00 ± 0.34	0.17 ± 0.02	0.14 ± 0. 0.23 ± 0.01		
Soil	0	–	–	67		0.015 ± 0.003 0.020 ± 0.003	–	–		
Grass	–	0.027 ± 0.003	–	–	0.16 ± 0.01	–	–	0.18 ± 0.01		
Marine ecosystem	32	0.052 ± 0.086	0.0025 ± 0.0004 0.33 ± 0.04	44	0.26 ± 062	0.005 ± .001 3.13 ± 0.22	0.14 ± 0.04	0.09 ± 0.02 0.22 ± 0.04	0.24 ± 0.17	0.057 ± 0.001 0.726 ± 0.013
Birds soft tissues	26	0.08 ± 0.12	0.0093 ± 0.0022 0.33 ± 0.04	70	0.30 ± 0.77	0.009 ± 0.002 3.13 ± 0.22	0.15 ± 0.05	0.09 ± 0.02 0.20 ± 0.04		
Birds bones	16	0.029 ± 0.040	0.0025 ± 0.0004 0.076 ± 0.009	21	0.15 ± 026	0.005 ± 0.001 0.55 ± 0.04	0.18 ± 0.04	0.14 ± 0.02 0.22 ± 0.04		
Other animals	0	–	–	9	–	0.07 ± 0.01	–	–		
Algae	67	0.032 ± 0.007	0.023 ± 0.005 0.041 ± 0.005	83	0.21 ± 0.12	0.023 ± 0.003 0.33 ± 0.03	0.123 ± 0.005	0.12 ± 0.01 0.13 ± 0.03		

The mean and standard deviation of ^238^Pu in marine samples is 0.052 ± 0.086 Bq/kg and ^239+240^Pu 0.26 ± 0.62. Results ranged 0.0025 ± 0.0004 ÷ 0.33 ± 0.04 Bq/kg and 0.005 ± 0.001 ÷ 3.13 ± 0.22 Bq/kg for ^238^Pu and ^239+240^Pu, respectively. Minimum value of ^238^Pu was detected in the *Pygoscelis adeliae* bones (AB9), maximal concentration in soft tissues of *Pagodroma nivea* (N3). The lowest value of ^239+240^Pu was found in bones of *Macronectes giganteus* (GB3), the highest in soft tissues of *Pagodroma nivea* (N3). In the algae group the average concentration is 0.032 ± 0.007 Bq/kg and 0.21 ± 0.12 Bq/kg for ^238^Pu and ^239+240^Pu respectively. In the group of birds’ bones, the average concentrations of ^238^Pu and ^239 + 240^Pu are 0.040 Bq/kg and 0.26 Bq/kg, respectively. Values vary from 0.0025 ± 0.0004 to 0.076 ± 0.009 Bq/kg (^238^Pu) and from 0.005 ± 0.001 to 0.55 ± 0.04 Bq/kg (^239+240^Pu). In the group of soft tissues mean concentration with a standard deviation of ^238^Pu is 0.08 ± 0.12 and ^239+240^Pu 0.30 ± 0.77 Bq/kg. For other organisms from the marine environment, the only value of ^239 + 240^Pu above the limit detection equals to 0.07 ± 0.01 Bq/kg, was discovered in the skin and fur of the *Mirounga leonina* (MT1). All results for ^238^Pu are below the detection limits in the latter sample group. These results obtained are comparable to the Antarctic radionuclides data published so far [2, 5, 18–20].

Among the terrestrial samples, the average values of ^238^Pu and ^239+240^Pu concentrations with their standard deviations are (for dry mass) 0.13 ± 0.14 Bq/kg and 0.62 ± 0.81 Bq/kg. Results vary from 0.0056 ± 0.0009 to 0.55 ± 0.10 Bq/kg (^238^Pu) and 0.0010 ± 0.0002 to 4.00 ± 0.34 Bq/kg (^239+240^Pu). The average concentration of ^238^Pu in mosses is 0.10 ± 0.13 Bq/kg and in lichens is 0.16 ± 0.14 Bq/kg and ^239+240^Pu: 0.47 ± 0.72 Bq/kg and 0.86 ± 0.91 Bq/kg for mosses and lichens respectively. Results range in mosses group is: 0.0058 ± 0.0009 ÷ 0.55 ± 0.10 Bq/kg for ^238^Pu and 0.010 ± 0.0002 ÷ 3.33 ± 0.31 ^239+240^Pu. Those numbers for lichens are: 0.021 ± 0.003 ÷ 0.55 ± 0.10 Bq/kg (^238^Pu) and 0.018 ± 0.003÷4.00 ± 0.34 Bq/kg (^239+240^Pu). Presented values are comparable with data published so far [4, 5, 18, 21].

**Fig. 2:**
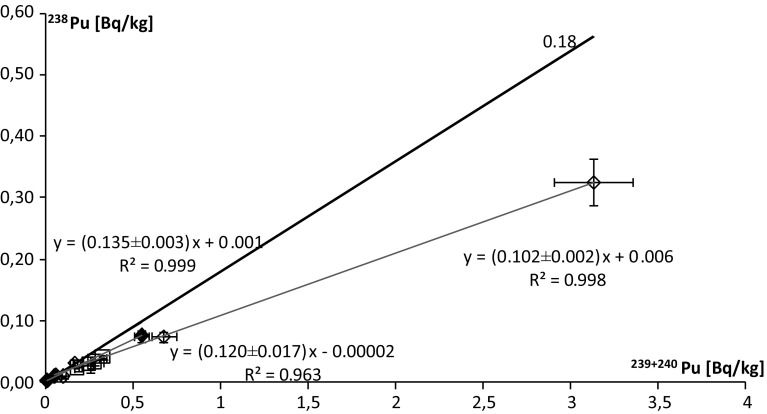
Correlation between ^238^Pu and ^239+240^Pu; open diamond marks for bird soft tissues and feathers, solid diamond marks for bird bones, open square marks for algae; black line represents global radioactive fallout ratio including SNAP 9 fallout

The determination of the ^238^Pu/^239+240^Pu activity ratio was possible in 13 marine samples and it was calculated directly from the counts associated with each signal in order to obtain smaller uncertainty values. The range of ^238^Pu/^239+240^Pu activity ratios vary from 0.09 ± 0.02 to 0.22 ± 0.04. The mean activity ratio for a set of samples can be estimated as the slope of linear regression. Thus, for algae mean ^238^Pu/^239+240^Pu is 0.120 ± 0.017, for bird soft tissues 0.102 ± 0.002 and for bird bones 0.135 ± 0.003 (Fig. 2). Correlations for all three groups are very high. In the whole collection of marine material, the linear regression slope is 0.103 ± 0.003 (Fig. 4). It should be noticed that the points corresponding to the bird soft tissue have the greatest impact on the result for the entire ecosystem. Nevertheless, the correlation between ^238^Pu and ^239+240^Pu in the whole group of marine samples is high, thus such trend is representative for the investigated marine material. The values of the ^238^Pu/^239+240^Pu activity ratio just described are lower than those presented by Jia et al. [2, 18], who received the samples from the marine environment (including water and sediments) 0.18 ÷ 0.29. Desideri et al. [19] published the average value 0.2 (together with terrestrial material). Those differences are higher than radioactive decay would cause. Mietelski et al. [5] estimated the mean ratio ^238^Pu/^239+240^Pu to be 0.136 by linear regression, but the *R*^2^ coefficient is very low (0.492).

**Fig. 3:**
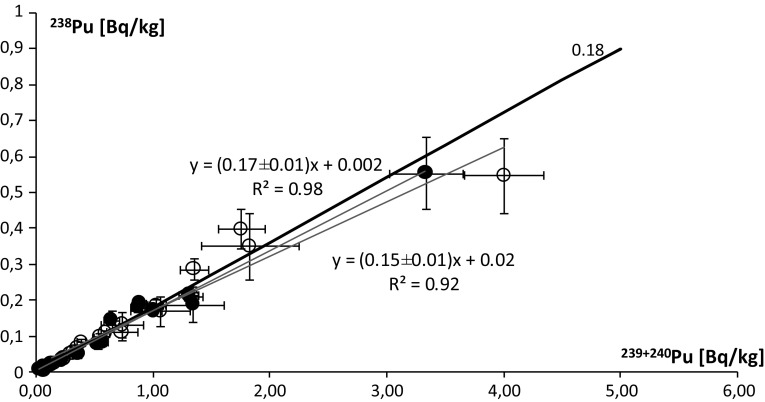
Correlation between ^238^Pu and ^239+240^Pu: solid dot marks for mosses, empty dot marks for lichens; the black line represents the global radioactive fallout ratio

The average activity ratio of ^238^Pu/^239+240^Pu in the terrestrial environment is 0.17 with its standard deviation of 0.03 (18%). It indicates a small differentiation of the plutonium activity ratio among the analyzed terrestrial samples. At the same time, the difference between extreme values is considerable; the range of results is 0.08 ± 0.01 ÷ 0.27 ± 0.05 (results obtained directly from the number of counts). The linear regression line fitted to the results of mosses has the slope 0.17 ± 0.01 and the *R*^2^ coefficient is high 0.98 (Fig. 3). The lichen regression line was plotted with a slightly weaker fit (*R*^2^ = 0.92), and its slope equals 0.15 ± 0.01 (Fig. 3). Therefore, the line plotted for the whole group of terrestrial ecosystem samples lies between these two lines (slope: 0.158 ± 0.006), and the correlation between ^238^Pu and ^239+240^Pu is high (*R*^2^ = 0.95). The results are similar to those published before [4, 5, 19, 21] and are consistent with global radioactive fallout ratio (including SNAP 9 re-entry) in the southern hemisphere [22].

**Fig. 4:**
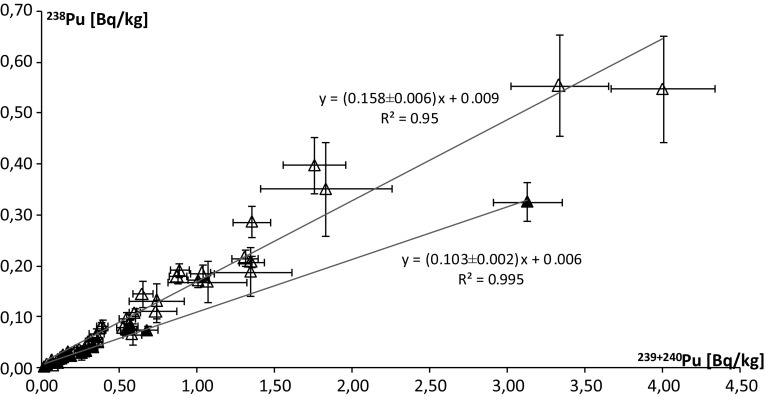
Correlation between ^238^Pu and ^239+240^Pu; open triangle marks for terrestrial samples, solid triangle marks for marine samples

These results indicate that there is a clear difference between terrestrial and marine ecosystems in terms of plutonium activity ratios (Fig. 4). Statistical analysis confirms this trend—at the 5% level of significance *p* = 0.0193, which indicates that the difference between marine and terrestrial Antarctic environments are statistically significant. A similar trend has already been suggested [5], but it was not so clear and was not statistically significant due to the small number of results. The interpretation of this difference is the hypothesis about the diversification of sources that affect the contamination of Antarctic ecosystems. On the one hand, marine organisms, mainly because of their migrations, have contact with sources characterized by more diverse plutonium ratios than those with which static terrestrial organisms interact. On the other hand, it cannot be ruled out that this difference is caused by the appearance, along with the sea currents in Antarctica, of water masses containing plutonium with slightly different isotopic ratios than those characterizing radioactive fallout in the past, which is suggested by results for non-migrant algae.

In samples from the marine environment, the average ^240^Pu/^239^Pu atom ratio is 0.24 and the standard deviation is 0.17 (69%). The range of the results obtained is high: 0.057 ± 0.001 ÷ 0.726 ± 0.013. Both extreme results relate to body fragments of the same individual, *Macronectes giganteus*. The smallest ratio was measured in feathers from the bird trunk (GT1), the largest in the bones of trunk (GB3). Another very high value (0.672 ± 0.014) was also noted in soft tissues from a trunk of the same individual (GT4). Such a large discrepancy in the plutonium atom ratios within the tissues of one individual may be due to its history. Plutonium embedded in bones and soft tissues is not subject to remobilization. Feathers, which are rebuilt every year, contain microelements from the current diet. The average ratio ^240^Pu/^239^Pu in marine samples without extreme values changes to 0.20 and the standard deviation drops significantly to 0.03.

The group of analyzed samples from the terrestrial ecosystem is characterized by an average ^240^Pu/^239^Pu atom ratio of 0.17 and a standard deviation of 0.01. These results are less varied than those obtained from the marine environment. The lowest value of the atom ratio, 0.147 ± 0.001, was measured in moss *Sanionia uncinata*; the highest ratio, 0.192 ± 0.001, was recorded in lichen *Usnea aurantiaco*-*atra*.

**Fig. 5:**
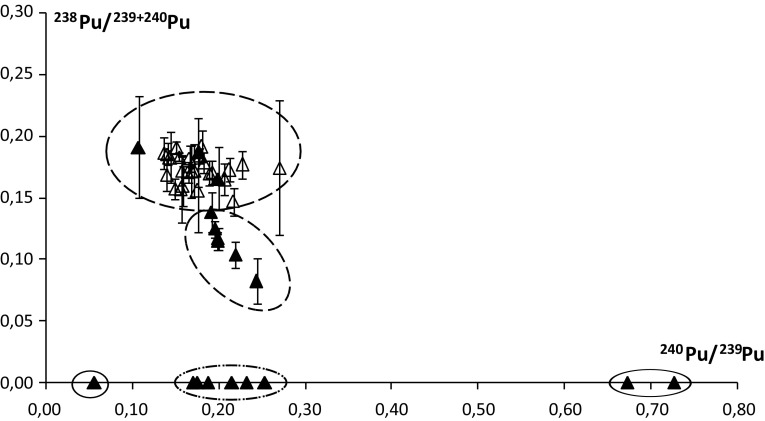
Relation between ^238^Pu/^239+240^Pu activity ratio and ^240^Pu/^239^Pu atom ratio; open triangle marks for terrestrial samples, solid triangles mark for marine samples; solid line-circled points represent samples of the same individual, *Macronectes giganteus*, while other circles (dashed line) indicate distinctive groups of different isotopic ratios

Figure 5 is presenting the relation between ^238^Pu/^239+240^Pu activity ratios and ^240^Pu/^239^Pu atom ratios, in which several groups can be distinguished. The first and main group consists of terrestrial samples and three samples from the marine environment (NB2, AB9, AT3). Their isotopic ratios are consistent with global fallout ratios characteristic to the southern hemisphere activity ratio 0.18 (with distinctive input of ^238^Pu from the SNAP-9A accident) and atom ratio 0.18 [22, 23]. Similar results were obtained in sub-ice-shelf sediments from the West Antarctic Ice Sheet [7]. The following group are marine samples with lower activity ratios, lower than 0.18 and with ^240^Pu/^239^Pu atom ratios in the same range as terrestrial samples. The next group comprises samples which are characterized by the lack of ^238^Pu (results below limits of detection) and the ^240^Pu/^239^Pu ratio similar to the two previous groups. There is no SNAP-9A influence evident and ^230+240^Pu comes from global radioactive fallout in this data set. It should be noted that this samples’ collection contains only animal specimens (algae are in the second group). Marine birds and mammals migrate over very long distances, which may be reflected in plutonium ratios. The next is a single point (GT1, circled solid line) also located on the *X*-axis with a much lower atom ratio than the previous group. Such a low ratio is interpreted as possible influence of plutonium from bursts and safety test sites on the atolls of Mururoa and Fangataufa in the French Polynesia region where the ^240^Pu/^239^Pu ratio is 0.03 ÷ 0.05 [24, 25]. In one of the layers of the glacial core from Eastern Antarctica (Dome C), the low ^240^Pu/^239^Pu ratio of 0.09 was measured [26], which authors identify with the French tests. It is possible, therefore, that animals living on areas around the South Pole have been in contact with a plutonium source of low (< 0.1) ^240^Pu/^239^Pu atom ratio. The last group consists of two points: GB3 and GT4 (circled solid line, too), coming from the same individual as GT1, *Macronectes giganteus*, in which no ^238^Pu was found (results below detection limits) and the atom ratios are much higher than in other samples. Laboratory contamination (there are no plutonium sources of such a high plutonium atom ratio in laboratories where measurements and radiochemical procedures were conducted) and statistical measuring errors or problems (there were very high numbers of counts for extreme samples, several thousands of counts for both masses when a detection limit is 200 counts) were excluded as an explanation for the higher ratios. There are no nuclear test sites characterized by such a high plutonium atom ratio. ^240^Pu is a daughter nuclide of ^244^Cm which is used in the analytical instrument (XRF spectrometer) for space applications [27]. However, there is no information about the loss or unsealing of such a generator in Antarctica. There is data on a spacecraft accident that had a ^244^Cm source on board—Russian Mars 96 spacecraft [27, 28]. It fell off the coast of Chile near the border with Bolivia and was not found so far. In addition, there were considerable quantities of ^238^Pu on board. In tissues of *Macronectes giganteus* ^238^Pu was not observed, but this could be explained by a more secure design of a thermogenerator than a spectrometer with ^244^Cm. However, if the excess of ^240^Pu in the samples would have come from the ^244^Cm decay, the traces of the latter (5762.6 keV; 5804.8 keV) would be seen in the Am spectra of these samples. Americium in these samples was analyzed according to procedure described by [14, 29] where curium is isolated along with the americium [14, 30]. Counts at ~ 5800 keV energy, although, were not observed. Therefore, it is difficult to find a reason for such high atom ratios (0.726 ± 0.013, 0.672 ± 0.014). One can only put forward a hypothesis that they are the result of incidental contamination by absorption which took place much earlier than the last year of the bird’s life, because the atom ratio measured in the outer feathers is radically different; molting occurs annually.

After removing GB3 and GT4 samples from the dataset (variances of marine and terrestrial data sets vary significantly) and applying the Welch test (unequal variances *t* test [31]), the differences between terrestrial and marine sample sat the level of 5% of significance, are statistically significant (*p* = 0.0122). This result confirms a distinct difference between the marine and terrestrial environment, which was observed on the basis of activity relations ^238^Pu and ^239+240^Pu.

## Conclusions

Thanks to the use of both alpha and mass spectrometry, the hypothesis about the influence of sources with different plutonium isotopic ratios on the Antarctic terrestrial and marine environment [5] was verified and confirmed. Differences between ecosystems showed statistical significance in the case of the ^238^Pu/^239+240^Pu activity ratios and were additionally confirmed by a statistically significant difference in the ^240^Pu/^239^Pu atom ratios. This is interpreted as the impact on the marine ecosystem of water masses characterized by diverse isotopic ratios and the contact of marine organisms with plutonium with different isotopic ratios in other parts of the world.

The use of ICP-MS revealed big difference in ^240^Pu/^239^Pu atom ratios of within one organism, *Macronectes giganteus*. This paradox was explained using assumption of various times of contamination and the lack of remobilization of plutonium for feathers. At the same time, all three results differ significantly from the atom ratio of global radioactive fallout (0.18). The lower atom ratio in outer feathers is interpreted as the possibility of birds contact with waters contaminated with a plutonium from safety tests carried out on Pacific sites (French Polynesia). The high ratios are not caused by contamination or errors made during the measurements, nor does not it originate from ^244^Cm (e.g. from a fluorescence spectrometer in the Russian Mars 96 probe) either. It is speculative that this is a result of an incidental absorption of e.g. a hot particle with an atom ratio of approx. 0.7, but it seems that a convincing explanation of these “exotic” atom ratios remains unknown.

## Electronic supplementary material

Below is the link to the electronic supplementary material.

Supplementary material 1 (PDF 316 kb)
