# Supplementary material for: Variations of plutonium isotopic ratios in Antarctic ecosystems
Source: J Radioanal Nucl Chem. 2018 Oct 29;318(3):1511–8. doi: 10.1007/s10967-018-6274-6 (PMC6267146; doi:10.1007/s10967-018-6274-6)
Supplement: Supplementary file 1 — Supplementary material 1 (PDF 316 kb) [file 10967_2018_6274_MOESM1_ESM.pdf]

Supplementary information

Table S1 Sampling sites with coordinates and sampling dates of lichens; KGI – King George Island

Sample	species	sample place	coordinates	sampling date
UA1	*Usnea antarctica*	Melville Peninsula (KGI)	62° 01' S<br>57° 40' W	12.2005
UA3	*Usnea antarctica*	Lions Rump (KGI)	62° 08' 20" S<br>58° 08' W	05.01.2007
UA4	*Usnea antarctica*	Carlini Base (KGI)	62º 07' 13'' S<br>58º 23' 46,4'' W	07.12.2005
UA6A	*Usnea aurantiaco-atra*	Hennequin Point (KGI)	62° 07' 13" S<br>58° 23' 46,6" W	16.02.2006
UA7A	*Usnea aurantiaco-atra*	Brown Station (AP)	64° 53' S<br>62° 53' W	12.12.2006
UA8A	*Usnea aurantiaco-atra*	Ferraz Station (KGI)	62° 05' 21,7" S<br>58° 23' 54,9" W	28.01.2007
UA9	*Usnea antarctica*	Machu Picchu Station (KGI)	62° 05' 20" S<br>58° 28' W	16.02.2006
UA10	*Usnea antarctica*	Blue Dyke (KGI)	62° 13' 30" S<br>58° 27' W	10.01.2006
UA11	*Usnea antarctica*	Keller Peninsula (KGI)	62° 05' S<br>58° 23' 30" W	11.03.2006
UA12	*Usnea antarctica*	Hennequin Point (KGI)	62° 07' 13" S<br>58° 23' 46,4" W	16.02.2006
UA13	*Usnea antarctica*	Vauréal Peak (KGI)	62º 10' 52,8'' S<br>58º 17' 32,3'' W	04.01.2007
UA14	*Usnea antarctica*	Moraines of Ecology Glacier (KGI)	62° 10' S<br>58° 25' W	14.02.2002
UA15A	*Usnea aurantiaco-atra*	Bellingshausen Station (KGI)	62° 11' 55,8" S<br>58° 57' 52,4" W	19.12.2005
UA16	*Usnea antarctica*	Penguin Island	62º 06' 15,6'' S<br>58º 59' 40,2'' W	15.02.2006
UA17A	*Usnea aurantiaco-atra*	Hennequin Point (KGI)	62º 07' 13'' S<br>58º 23' 46,4'' W	26.12.2005
UA18	*Usnea antarctica*	Marsh Station (KGI)	62º 11' 39,5'' S<br>58º 58' 35'' W	17.12.2005
UA20	*Usnea antarctica*	Brown Station (AP)	64° 53' S<br>62° 53' W	12.12.2006
UA21A	*Usnea aurantiaco-atra*	Kapitan Peak (KGI)	62° 05' 30" S<br>58° 28' W	16.02.1980
UA22A	*Usnea aurantiaco-atra*	Red Hill (KGI)	62° 14' S<br>58° 30' W	26.01.2002
UA23	*Usnea antarctica*	Red Hill (KGI)	62° 14' S<br>58° 30' W	26.01.2002
UA24	*Usnea antarctica*	Brown Station (AP)	64° 53' S<br>62° 53' W	12.12.2006

Table S2 Species, sampling sites with coordinates and sampling dates of mosses; KGI – King George Island, AP – Antarctic Peninsula

sample	sample place	coordinates	sampling date
SU1	Penguin Island	62º05’45,5” S<br>57º55’49,7” W	01.01.2007
SU4	Great Wall<br>(KGI)	62º18’07” S<br>58º57’33,8” W	16.02.2005
SU7	Three Sister Point, Olech Hills<br>(KGI)	62º04’S<br>57º55’ W	10.01.2007
SU8	Bellingshausen Station<br>(KGI)	62°11’ 56” S<br>58° 57’ 52” W	19.12.2005
SU12	Penguin Island	62° 05 45,5”S<br>57° 55’ 49,7” W	1.01.2007
SU14	Deception Island	62° 57’ S<br>60° 38’ W	10.02.2006
SU16	Puchalski Peak<br>(KGI)	62° 10’ S<br>58° 17’ 30”W	12.2005
SU18	Marsh Station<br>(KGI)	62º11’18,5” S<br>58º59’56,2” W	03.01.2007
SU20	Hennequin Point<br>(KGI)	62º07’13” S<br>58º23’46,4” W	26.12.2005
SU25	Hennequin Point<br>(KGI)t	62° 07’ 13”S<br>58° 23’ 46,4” W	16.02.2006
SU28	Deception Island	62° 57’ S<br>60° 38’ W	01.02.2006
SU29	Marsh Station<br>(KGI)	62° 11’ 18,5” S<br>58° 59’ 56,2” W	18.12.2005
SU30	Blue Dyke<br>(KGI)	62º13’30” S<br>58º27’ W	10.01.2006
SU32	Lions Rump<br>(KGI)	62° 08’ 20” S<br>58° 08’ W	05.01.2007
SU33	Moraines of Ecology Glacier<br>(KGI)	62° 10’ S<br>58° 25’ W	14.02.2002
SU34	Vauréal Peak<br>(KGI)	62°10’52,8” S<br>58°17’32,3” W	04.01.2007
SU35	Penguin Island	62° 06’ 15,6” S<br>57° 55’ 40,2” W	31.12.2006
SU36	Turret Point<br>(KGI)	62° 04’ 55,7” S<br>57° 56’ 50,2” W	23.01.2009
SU37	Carlini Base<br>(KGI)	62°15’30,8” S<br>58°37’92,3” W	06.02.2006
SU38	Penguin Island	62° 05’ 58” S<br>57° 55’ 19,7” W	1.01.2007
SU39	Machu Picchu Station<br>(KGI)	62° 05’ 20” S<br>58° 28’ W	08.01.2002
SU40	Red Hill<br>(KGI)	62° 14’ S<br>58° 30’ W	26.01.2002
SU41	Moraines of Ecology Glacier<br>(KGI)	62º10’0,55” S<br>58º27’64,2” W	14.03.2015
SU42	Vauréal Peak<br>(KGI)	62° 10’ 45” S<br>58° 17’ 30” W	24.01.2002
SU43	Moraines of Ecology Glacier	62° 10’ S	14.02.2002

	(KGI))	58° 25’ W	
SU44	Peter I Island	68° 51’ S<br>90° 37’ W	12.12.2006
SU45	Komandor Peak<br>(KGI)	62° 06’ S<br>58° 29’ W	21.12.2007

Table S3 Species, sampling sites with coordinates and sampling dates of other terrestrial samples; KGI – King George Island

Sample	species/description	sample place	coordinates	sampling date
D1	*Deschampsia antarctica,* grass	Penguin Island	62° 05’ 48,2” S<br>57° 55’ 33,8” W	26.12.2006
S1	initial soil	Moraines of Ecology Glacier (KGI)	62 10 15 S<br>58 28 20,9 W	14.03.2015
S2	initial soil	Moraines of Ecology Glacier (KGI)	62 10 12,6S<br>58 28 17,1W	13.03.2015
S3	initial soil	Moraines of Ecology Glacier (KGI)	62 10 15 S<br>58 28 20,9 W	14.03.2015

Table S4 Species, sampling sites with coordinates and sampling dates of Antarctic birds; KGI – King George Island

Sample	species/description	sample place	coordinates	sampling date
AB1 & AT1	*Pygoscelis adeliae* (head)	Patelnia (KGI)	62° 14' S<br>58° 28' W	10.01.2006
AB2 & AT2	*Pygoscelis adeliae* (paws)			
AT3	*Pygoscelis adeliae* (feathers and skin)			
AB4 & AT4	*Pygoscelis adeliae* (skeleton)			
AB5	*Pygoscelis adeliae*	Penguin Ridge (KGI)	62° 08' 30" S<br>58° 29' W	22.02.2010
AB6 & AT6	*Pygoscelis adeliae*	Jasnorzewski Gardens (KGI)	62° 09' 45,8" S<br>58° 27' 50,3" W	3.02.2010
AB7 & AT7	*Pygoscelis adeliae*	Jasnorzewski Gardens (KGI)	62° 09' 45,8" S<br>58° 27' 50,3" W	3.02.2010
AB8 & AT8	*Pygoscelis adeliae*(skeleton with remains of skin)	Jasnorzewski Gardens (KGI)	62° 09' 45,8" S<br>58° 27' 50,3" W	3.02.2010
AB9	*Pygoscelis adeliae* (skeleton)	Jasnorzewski Gardens (KGI)	62° 09' 45,8" S<br>58° 27' 50,3" W	15.02.2002
CB1	*Cathatacta antarctica* (wing)	Jasnorzewski Gardens (KGI)	62° 09' S<br>58° 28' 30" W	04.01.2015
CB2 & CT2	*Cathatacta antarctica* (skeleton with remains of skin)	Bellingshausen Station (KGI)	62° 11" 55,8" S<br>58° 57' 52,4" W	19.12.2005
CB3 & CT3	*Cathatacta antarctica*	Ubocz (KGI)	62° 10' S<br>58° 29' W	9.01.2006
CB4 & CT4	*Cathatacta antarctica* (wing)	Turret Point (KGI)	62° 04' 55,7" S<br>57° 56' 50,2" W	22.02.2009
GT1	*Macronectes giganteus* (feathers of trunk)	Moraines of Ecology Glacier (KGI)	62° 10' S<br>58° 25' W	09.01.2006
GB2 & GT2	*Macronectes giganteus* (head)			
GB3 & GT3	*Macronectes giganteus* (wings and paws)			
GB4 & GT4	*Macronectes giganteus* (trunk)			
GB5& GT5	*Macronectes giganteus*	Penguin Island	62° 05' 58" S<br>57° 55' 19,7" W	1.02.2010
NB1 & NT1	*Pagodroma nivea* (wings)	Schirmacher Oasis	70° 45' 2,4" S<br>11° 32' 33,7" E	21.01.2004
NB2 & NT2	*Pagodroma nivea* (wing)	Schirmacher Oasis	70° 45' 2,4" S<br>11° 32' 33,7" E	21.01.2004
N3	*Pagodroma nivea* (wing)	Schirmacher Oasis	70° 45' 2,4" S<br>11° 32' 33,7" E	21.01.2004
NB4	*Pagodroma nivea* (bones)	Schirmacher Oasis	70° 45' 15,1" S<br>11° 32' 55,3" E	21.01.2004
PF1	*Pygoscelis papua* (feathers after molting)	Moraines of Ecology Glacier (KGI)	62° 10,5' S<br>58° 25' W	20.02.2006
PF2	*Pygoscelis papua* (feathers after molting)	Moraines of Ecology Glacier (KGI)	62° 10,5' S<br>58° 25' W	06.02.2007
PF3	*Pygoscelis papua* (feathers after molting)	Moraines of Ecology Glacier (KGI)	62° 10,5' S<br>58° 25' W	22.02.2002
PE1	*Pygoscelis adeliae* (eggs shells)	Moraines of Ecology Glacier (KGI)	62° 10,5' S<br>58° 25' W	22.02.2010
PE2	*Pygoscelis adeliae* (eggs shells)	Moraines of Ecology Glacier (KGI)	62° 10,5' S<br>58° 25' W	29.12.2008
PE4	*Pygoscelis adeliae* (eggs shells)	Jasnorzewski Gardens (KGI)	62° 09' 45,8" S<br>58° 27' 50,3" W	22.02.2010

Table S5 Species, sampling sites with coordinates and sampling dates of other marine organisms; KGI – King George Island

sample	species/description	sample place	coordinates	sampling date
B1	*Himantothallus grandifolius*	Shag Point (KGI)	62° 09'20" S 58° 27' 30" W	20.12.2006
B2	*Himantothallus grandifolius*	Shag Point (KGI)	62° 09'20" S 58° 27' 30" W	20.12.2006
B3	*Himantothallus grandifolius*	Shag Point (KGI)	62° 09'20" S 58° 27' 30" W	20.12.2006
B7	*Iridaea cordata*	Shag Point (KGI)	62° 09'20" S 58° 27' 30" W	20.12.2006
B8	*Iridaea cordata*	Shag Point (KGI)	62° 09'20" S 58° 27' 30" W	20.12.2006
B9	*Iridaea cordata*	Shag Point (KGI)	62° 09'20" S 58° 27' 30" W	20.12.2006
H1	*Harpagifer antarcticus* (head)	Admirality Bay		08.02.2002
CH1	*Chaenocephalus aceratus* (tail)	Admirality Bay		8.02.2002
NM1 & NS1	*Nacella concinna* (meat and shells)	Arctowski Station (KGI)	62° 09' 20" S 58° 27' 30" W	10.02.2009
MT1	*Mirounga leonina* (skin fragment)	Demay Point (KGI)	62° 11' S 58° 25' 30" W	21.01.2007
WT1	*Leptonychotes weddellii* (skin fragment)	Jasnorzewski Gardens (KGI)	62° 09' 42" S 58° 28' 10" W	31.12.2008
WB2 & WT2	*Leptonychotes weddellii* (bone with skin fragment)	Jasnorzewski Gardens (KGI)	62° 09' 42" S 58° 28' 10" W	31.12.2008

Table S6 Activity concentrations of $^{239+240}Pu$, ^{238}Pu in Bq/kg ash weight, activity ratios $^{238}Pu/^{239+240}Pu$ calculated directly from counts number and atom ratios for marine samples

sample name	$^{239+240}Pu$	^{238}Pu	$^{238}Pu/^{239+240}Pu$	$^{240}Pu/^{239}Pu$
B1	0.33±0.03	0.041±0.005	0.12±0.01	0.195±0.001
B2	0.28±0.02	0.033±0.005	0.12±0.01	0.199±0.001
B3	0.25±0.05	0.029±0.012	0.12±0.01	0.198±0.002
B7	0.18±0.02	0.023±0.005	0.13±0.03	-
B8	0.02±0	<0.005	-	-
B9	<0.02	<0.03	-	-
H1	<0.034	<0.03	-	-
CH1	<0.051	<0.04	-	-
MT1	0.07±0.01	<0.01	-	0.188±0.004
NM1	<0.014	<0.016	-	-
NS1	<0.0004	<0.0003	-	-
WB2	<0.006	<0.007	-	-
WT1	<0.058	<0.02	-	-
WT2	<0.055	<0.05	-	-
PE1	<0.001	<0.001	-	-
PE2	<0.001	<0.001	-	-
PE4	<0.002	<0.002	-	-
AB1	<0.01	<0.008	-	-
AB2	<0.01	<0.005		-
AB4	<0.002	<0.002	-	0.176±0.004
AB5	<0.004	<0.001	-	-
AB6	<0.002	<0.002	-	-
AB7	<0.006	<0.006	-	-
AB8	<0.002	<0.002	-	-
AB9	0.01±0	0.002±0	0.22±0.04	0.107±0.001
CB1	<0.01	<0.013	-	-
CB2	<0.002	<0.003	-	-
CB3	<0.007	<0.008	-	0.726±0.013
CB4	<0.013	<0.014	-	-
GB2	<0.01	<0.006	-	-
GB3	0.005±0.001	<0.002	-	-
GB4	<0.002	<0.002	-	-
GB5	<0.06	<0.04	-	-
NB1	<0.03	<0.021	-	-
NB2	0.05±0	0.009±0.001	0.18±0.03	0.199±0.003
NB4	0.55±0.04	0.076±0.009	0.14±0.02	0.191±0.001
AT1	0.17±0.02	<0.05	-	0.17±0.003
AT2	0.16±0.02	<0.04	-	-
AT3	0.05±0	0.01±0.001	0.19±0.03	0.176±0.003
AT4	0.03±0	<0.012	-	-
AT5	<0.008	<0.009	-	-
AT6	<0.006	<0.007	-	-
AT7	0.01±0	<0.006	-	-
AT8	0.17±0.02	0.033±0.005	0.19±0.03	0.17±0.002
CT1	<0.019	<0.02	-	-
CT2	0.03±0.01	<0.019	-	0.254±0.004

CT3	0.06±0.01	0.013±0.002	0.20±0.04	-
CT4	0.025±0.005	<0.02	-	-
GT1	0.02±0	<0.008	-	0.057±0.001
GT2	<0.015	<0.015	-	-
GT3	0.01±0	<0.005	-	-
GT4	0.02±0	<0.004	-	0.672 ±0.014
GT5	0.08±0.01	<0.012	-	0.232±0.005
N3	3.13±0.22	0.326±0.038	0.10±0.01	0.219±0.002
NT1	0.1±0.01	0.009±0.002	0.09±0.02	0.244±0.002
NT2	0.68±0.07	0.074±0.008	0.11±0.01	0.215±0.002
PF1	<0.038	<0.03	-	-
PF2	<0.073	<0.06	-	-
PF3	<0.095	<0.09	-	-

Table S7 Activity concentrations of $^{239+240}Pu$, ^{238}Pu in Bq/kg dry weight, activity ratios $^{238}Pu/^{239+240}Pu$ calculated directly from counts number and atom ratios for terrestrial samples.

sample name	$^{239+240}Pu$	^{238}Pu	$^{238}Pu/^{239+240}Pu$	$^{240}Pu/^{239}Pu$
SU1	1.31±0.09	0.21±0.02	0.16±0.01	0.171±0.004
SU4	3.33±0.31	0.51±0.1	0.17±0.01	0.182±0.001
SU7	0.046±0.005	<0.007	-	-
SU8	0.22±0.02	0.034±0.006	0.16±0.01	0.172±0.007
SU12	0.57±0.05	0.083±0.011	0.15±0.02	-
SU14	<0.012	<0.01	-	-
SU16	0.88±0.06	0.19±0.01	0.22±0.01	0.147±0.001
SU18	0.06±0.01	0.010±0.002	0.16±0.03	
SU20	0.52±0.05	0.079±0.013	0.15±0.01	0.158±0.003
SU25	0.65±0.07	0.14±0.02	0.22±0.01	-
SU28	0.0010±0.0002	<0.0008	-	-
SU29	0.13±0.01	<0.01	-	-
SU30	0.068±0.005	0.0056±0.0009	0.08±0.01	-
SU32	0.060±0.006	0.014±0.002	0.23±0.04	-
SU33	0.06±0.01	<0.02	-	-
SU34	0.13±0.01	0.022±0.003	0.17±0.02	-
SU35	0.10±0.01	0.016±0002	0.16±0.02	-
SU36	0.12±0.01	0.019±0.002	0.17±0.02	0.16±0.001
SU37	0.13±0.01	0.022±0.003	0.17±0.02	0.172±0.003
SU39	0.87±0.06	0.18±0.01	0.21±0.01	0.165±0.001
SU40	0.13±0.01	0.023±0.004	0.18±0.03	0.156±0.001
SU41	0.013±0.002	<0.005	-	-
SU42	0.025±0.003	0.0069±0.0012	0.27±0.05	0.175±0.003
SU43	1.00±0.07	0.17±0.01	0.17±0.01	-
SU44	1.34±0.26	0.19±0.05	0.14±0.01	0.168±0.001
SU45	0.36±0.03	0.052±0.008	0.14±0.02	0.183±0.003
UA1	0.59±0.04	0.11±0.01	0.18±0.01	0.179±0.002
UA3	0.35±0.05	0.07±0.01	0.19±0.01	0.17±0.003
UA4	0.24±0.02	0.037±0.004	0.16±0.02	-
UA6A	1.03±0.08	0.19±0.02	0.18±0.01	0.192±0.001

UA7A	0.54±0.05	0.097±0.011	0.18±0.01	-
UA8A	1.75±0.2	0.40±0.06	0.23±0.01	0.177±0.002
UA9	0.13±0.01	0.021±0.003	0.16±0.03	0.157±0.003
UA10	0.30±0.02	0.052±0.006	0.18±0.02	-
UA11	1.35±0.12	0.29±0.03	0.21±0.01	0.173±0.002
UA12	<0.03	<0.03	-	-
UA13	0.39±0.04	0.08±0.01	0.21±0.01	-
UA14	0.018±0.003	<0.006	-	-
UA15A	0.36±0.03	0.050±0.09	0.14±0.01	0.186±0.001
UA16	0.74±0.18	0.13±0.03	0.18±0.01	0.184±0.001
UA17A	1.06±0.26	0.17±0.04	0.16±0	-
UA18	0.73±0.13	0.11±0.02	0.15±0.01	0.191±0.001
UA20	0.15±0.01	0.025±0.004	0.16±0.02	-
UA21A	4.00±0.34	0.55±0.10	0.14±0.01	0.187±0.002
UA22A	1.35±0.08	0.21±0.01	0.15±0	0.184±0.003
UA23	1.83±0.42	0.35±0.09	0.19±0.01	0.171±0.003
UA24	0.31±0.02	0.053±0.006	0.16±0.02	-
D1	0.16±0.01	0.027±0.003	0.17±0.02	0.172±0.001
S1	0.015±0.003	<0.014	-	-
S2	0.020±0.003	<0.012	-	-
S3	<0.008	<0.011	-	-
